# *OsGRF4^AA^* compromises heat tolerance of developing pollen grains in rice

**DOI:** 10.3389/fpls.2023.1121852

**Published:** 2023-02-22

**Authors:** Yujian Mo, Guangyan Li, Li Liu, Yingjie Zhang, Junyi Li, Meizhen Yang, Shanlan Chen, Qiaoling Lin, Guanfu Fu, Dianfeng Zheng, Yu Ling

**Affiliations:** ^1^ College of Coastal Agricultural Sciences, Guangdong Ocean University, Zhanjiang, China; ^2^ South China Branch of National Saline-Alkali Tolerant Rice Technology Innovation Center, Zhanjiang, China; ^3^ State Key Laboratory of Rice Biology, China National Rice Research Institute, Hangzhou, China

**Keywords:** *OsGRF4*, rice, heat tolerance, carbohydrate metabolism, gene transcription, pre-mRNA alternative splicing

## Abstract

Extreme high temperature at the meiosis stage causes a severe decrease in spikelet fertility and grain yield in rice. The rice variety grain size on chromosome 2 (GS2) contains sequence variations of *OsGRF4* (*Oryza sativa* growth-regulating factor 4; *OsGRF4^AA^*), escaping the microRNA miR396-mediated degradation of this gene at the mRNA level. Accumulation of *OsGRF4* enhances nitrogen usage and metabolism, and increases grain size and grain yield. In this study, we found that pollen viability and seed-setting rate under heat stress (HS) decreased more seriously in GS2 than in its comparator, Zhonghua 11 (ZH11). Transcriptomic analysis revealed that, following HS, genes related to carbohydrate metabolic processes were expressed and regulated differentially in the anthers of GS2 and ZH11. Moreover, the expression of genes involved in chloroplast development and photosynthesis, lipid metabolism, and key transcription factors, including eight male sterile genes, were inhibited by HS to a greater extent in GS2 than in ZH11. Interestingly, pre-mRNAs of *OsGRF4*, and a group of essential genes involved in development and fertilization, were differentially spliced in the anthers of GS2 and ZH11. Taken together, our results suggest that variation in *OsGRF4* affects proper transcriptional and splicing regulation of genes under HS, and that this can be mediated by, and also feed back to, carbohydrate and nitrogen metabolism, resulting in a reduction in the heat tolerance of rice anthers.

## 1 Introduction

Heat stress (HS) is an abiotic stress that causes severe loss in rice production (Tang et al., 2020; Qu et al., 2022; Shen et al., 2022). What is worse, the Intergovernmental Panel on Climate Change (IPCC) recently predicted that the mean temperature on Earth will continuously increase to 1.5–2°C above pre-industrial averages by 2050, even if net zero CO_2_ emissions were to be achieved immediately (Shukla et al., 2022).

Developing pollen grains are more vulnerable to high temperatures than cells from other plant tissues. High temperatures cause production loss directly by decreasing pollen grain viability and seed formation in crops (Draeger and Moore, 2017; Peng et al., 2020; Bokshi et al., 2021; Naranjo et al., 2022). As a key modulator of HS response, heat stress transcription factor A2 (HSFA2) plays an essential role in the thermotolerance of meiocytes and microspores in plants (Frank et al., 2009; Akerfelt et al., 2010; Vihervaara et al., 2013; Fragkostefanakis et al., 2016). In tomato, male meiocytes and microspores are susceptible to HS owing to their inefficient regulatory response to increasing temperature. This sensitivity could be partially mitigated by enhanced expression of *HSFA2* and several other HS-responsive genes (Hedhly et al., 2016). Carbohydrate metabolites, energy status, and phytohormone abundance could also alter the thermotolerance of microspores and anthers of plants (Jambhekar and Amon, 2008; Hedhly et al., 2016; Smith and Zhao, 2016). In the grain sorghum (*Sorghum bicolor*), HS decreased cell wall invertase (CWI)-mediated sucrose hydrolysis in microspores and anthers, leading to altered carbohydrate metabolism and starch deficiency in pollen grains (Jain et al., 2010). A transient HS treatment at the tetrad stage of maize caused significant regulation of starch, lipid, and energy biosynthesis-related genes. Correspondingly, increased levels of sucrose and its monosaccharide components, and decreased levels of pyruvate, fatty acids, and saturated fatty acids, were found in plants grown under HS (Begcy et al., 2019). Similarly, differential expression changes in chlorophyll- and photosynthesis-related genes, together with a disturbance of carbohydrates, were found in heat-treated rice anthers (Liu et al., 2020). In rice, salicylic acid may increase the HS tolerance of pollen mother cells through hydrogen peroxide (H_2_O_2_)-mediated signaling pathways (Feng et al., 2018) and catalase (CAT)-mediated detoxification of reactive oxygen species (ROS) (Zhao et al., 2018; Gong et al., 2019). Furthermore, the exogenous application of abscisic acid prior to HS enhanced sucrose transport and accelerated sucrose metabolism to maintain the carbon balance and energy homeostasis, which consequently mitigated the heat damage to anthers (Rezaul et al., 2019).

Growth regulation factors (GRFs) are a group of plant-specific transcription factors. The N-terminal regions of the GRFs contain two highly conserved domains, the QLQ and WRC domains. The QLQ domain serves as a protein–protein interacting interface, and the WRC domain functions as the DNA-binding site of the protein (Kim and Tsukaya, 2015). Recently, many studies have demonstrated that expression levels of both *OsGRF4* and other GRF genes are post-transcriptionally regulated by the microRNA miR396 (Liu et al., 2014; Che et al., 2015; Duan et al., 2015; Li S. et al., 2016; Liebsch and Palatnik, 2020; Rossmann et al., 2020; Lu et al., 2021). Therefore, several rice varieties possessing, either naturally or artificially, a sequence variation on *OsGRF4* (*Oryza sativa* growth-regulating factor 4; *OsGRF4^AA^*) that blocks the binding site of miR396 accumulate more *OsGRF4* transcripts and proteins (Hu et al., 2015). These rice varieties promote and integrate nitrogen assimilation, carbon fixation, and cellular growth, then increase grain size and weight by significantly up-regulating expression levels of *Adenine Methyltransferase 1.1 (AMT1.1)*, *glutamine synthetase 1.2 (GS1.2)*, *glutamine synthetase 2 (GS2)*, and *NADH-glutamate synthase 2 NADH–GOGAT2* (Li et al., 2018). Regulation of the expression of *OsGRF4* by miR396 has been shown to be essential for grain production in rice under nitrogen-deficient conditions (Zhang et al., 2020).

The GRF-mediated regulation of growth and stress response in plants usually requires the cooperation of other regulators. A transcription co-activator, GRF-interacting factor (GIF), modulates the growth of both vegetative and reproductive organs in rice (Duan et al., 2015; He et al., 2017), which can be mediated by gene transcriptional activation, which in turn is controlled by the GRF–GIF combination (Li S. et al., 2016). Similar cooperation between GRF and GIF has also been found in other plants (Li S. et al., 2016; Lee et al., 2018). Furthermore, OsGRF4-mediated up-regulation of brassinosteroid signaling genes and MYB61, a transcription factor relevant to cellulose accumulation, is also necessary for the promotion of grain development (Che et al., 2015; Gao et al., 2020).

As enhanced expression of *OsGRF4* in a gibberellic acid-independent manner has been shown to overcome the disadvantage of low nitrogen use efficiency (NUE) of green revolution varieties (Li et al., 2018), variations in *OsGRF4* are considered a target for rice breeding in order to increase production (Liebsch and Palatnik, 2020; Lu et al., 2021). However, whether and how the variation of *OsGRF4* affects stress tolerance in rice has not yet been studied. In this study, we compared the effects of HS on two rice genotypes, Zhonghua 11 (ZH11) and its *OsGRF4^AA^* near-isogenic line, grain size on chromosome 2 (GS2). Our experiment demonstrated that HS treatment at the meiotic stage caused a greater abundance of dead pollen grains and less seed setting in GS2 than in ZH11. Transcriptomic analysis demonstrated that a group of genes involved in abiotic stimuli and those relating to chloroplast and photosynthesis, including eight male sterile genes (MSGs), were, generally, inhibited more significantly by HS in anthers from GS2, which was consistent with the more serious heat damage to anthers and pollen grains in GS2. Furthermore, pre-mRNA alternative splicing (AS) analysis revealed differential AS regulation between the two genotypes in a considerable number of genes, including several genes that are important for meiosis development and the stress response, such as *CIRCADIAN CLOCK ASSOCIATED 1 (CCA1)*, *OsSPO11-5*, *HSFB2c*, and, interestingly, *OsGRF4* itself, suggesting that OsGRF4 affects the heat tolerance of rice anthers at both the transcription and pre-mRNA splicing levels.

## 2 Materials and methods

### 2.1 Plant materials, growth conditions, and treatments

The rice seeds of ZH11 and its *OsGRF4^AA^* near-isogenic line, GS2, were provided by Dr. Jiang Hu from the State Key Laboratory of Rice Biology, China National Rice Research Institute (Hu et al., 2015). The seeds were first grown in the China National Rice Research Institute (Hangzhou, China, 119°57′E, 30°03′N) under natural conditions until the pollen mother cell meiotic stage (Feng et al., 2018). Thereafter, the rice plants were divided into two groups and moved into separate transparent growth chambers with a relative humidity of around 70%–80% and natural sunlight conditions. Group 1 was subjected to a HS condition (i.e., 28°C from 16:01 to 8:59, and 38°C from 9:00 to 16:00), whereas the other group was grown under a normal temperature condition (i.e., 23°C from 16:01 to 8:59 and 28°C from 9:00 to 16:00). The treatment time was 6 days. Anthers were sampled at 16:00 on the last day of the treatment. Sampled anthers were immediately submerged in liquid nitrogen for 1 min and stored at –80°C until RNA extraction and sequencing library preparation.

### 2.2 Pollen viability measurement

Pollen viability was determined using the method described by Feng et al. (2018). Briefly, mature pollen grains were removed from the anthers of the florets, and placed on a glass slide with a drop of potassium iodide/iodine (KI/I_2_) solution. The slide was observed using a light microscope (DM4000B; Leica, Wetzlar, Germany). Eight replicates were investigated and photographed for the measurement of pollen viability.

### 2.3 Seed-setting rate measurement

Mature rice plants and panicles were photographed for phenotyping. Then the number of filled grains (FG) and abortive grains (AG) per panicle were measured. The seed-setting rate was calculated as FG/(FG + AG) × 100%.

### 2.4 RNA-sequencing and data analysis

RNA sequencing (RNA-Seq) and primary data analysis were performed at Shanghai Majorbio Bio-pharm Biotechnology Co., Ltd. (Shanghai, China). Total RNA was isolated from rice anthers using a plant RNA purification reagent (Invitrogen, California, USA). The quality and integrity of the RNA were measured by an Agilent Bioanalyzer 2100 system (Agilent Technologies, USA) and only high-quality RNA samples [OD_260/280_ = 1.8–2.2, OD_260/230_ ≥ 2.0, RNA integrity number (RIN) ≥ = 8.0, 28S : 18S ≥ 1.0, > 1 μg] were used to construct the sequencing library. RNA-Seq libraries were sequenced on an Illumina NovaSeq6000 platform to generate high-quality paired-end reads following the Illumina manufacturer’s recommendations. Next, data processing for differentially expressed gene (DEG) analysis was carried out using Kallisto (Bray et al., 2016) and 3D RNA-Seq App (Guo et al., 2021), with default settings (adjusted *p*-value < 0.01 and absolute log_2_-fold change > 1) unless specifically indicated otherwise. Pre-mRNA splicing analysis was performed using rMATS (replicate multivariate analysis of transcript splicing) (Shen et al., 2014). RNA read coverages were visualized using the program Integrative Genomics Viewer (IGV) (http://www.igv.org) by Rice Annotation Project (RAP) International Rice Genome Sequencing Project (IRGSP) 1.0 genome (https://rapdb.dna.affrc.go.jp/) as the reference genome.

### 2.5 qRT-PCR and RT-PCR

Reverse transcription-PCR (RT-PCR) and reverse transcription-quantitative PCR (qRT-PCR) were slightly modified compared with the methods previously described in (Chen et al., 2022). DNA digestion of total RNA samples was performed after RNA extraction by using an RNase-Free DNase Set (Vazyme, Catalog No. R223-01). Total RNA was then reverse transcribed using a SuperScript First-Strand Synthesis System to generate the first-strand cDNA libraries. qPCR was performed using Power SYBR Green PCR Master Mix (Yeasen Biotech; Catalog No. 11201ES08) under the following conditions: 95°C for 5 min, then cycles of 95°C for 15 s and 60°C for 1 min. The validation was assessed with three biological replicates for each sample. An actin gene (*Os03g0718150*) was used as the internal control for normalization of qPCR. The fold change was evaluated using the standard 2^(−ΔΔCT)^ method. Statistical analysis of gene expression in qRT-PCR was performed using Microsoft Excel 2013. In RT-PCR validation of AS events, primers flanking different exons were used. The PCR program was as follows: initial denaturation for 3 min at 95°C, followed by 40 cycles at 95°C for 30 s, 56°C for 30 s, and 72°C for 2 min. The final extension at 72°C lasted 5 min.

## 3 Results

### 3.1 Phenotypes of pollen grains and seed-setting of GS2 and ZH11 under different temperature conditions

**Figure 1:**
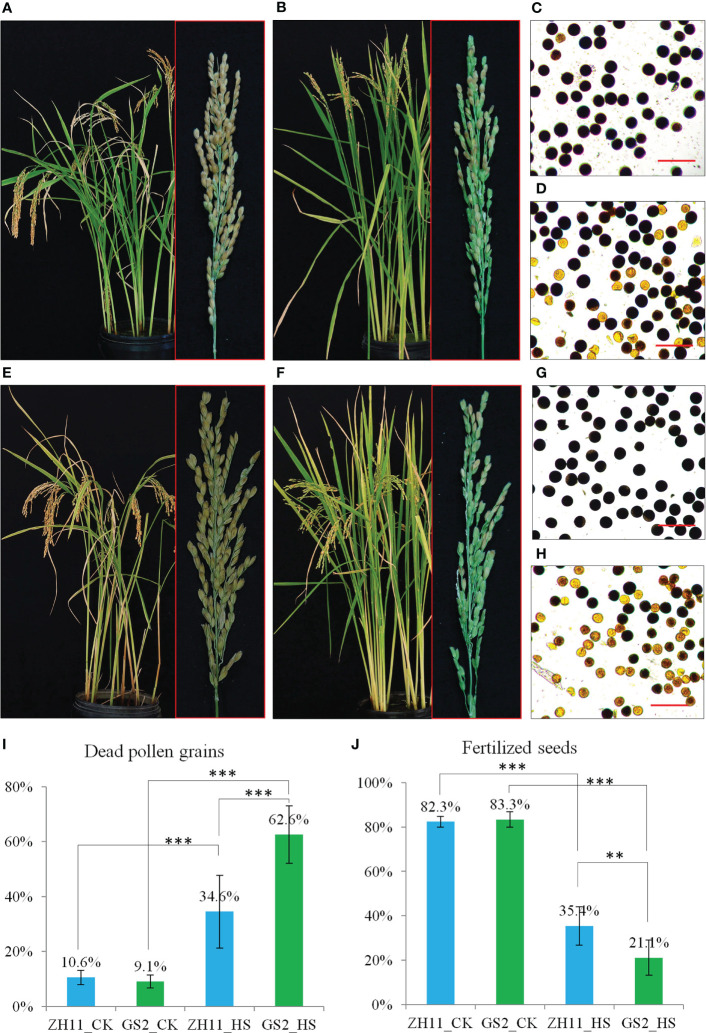
Phenotypes of Zhonghua 11 (ZH11) and grain size on chromosome 2 (GS2) under different temperature conditions at the pollen mother cell meiosis stage. **(A–D)** Photographs of whole plants, seed setting, and pollen grains in ZH11 with and without HS treatment. **(A, C)** Without heat stress (HS) treatment. **(B, D)** After HS treatment. **(E–H)** Photographs of whole plants, seed setting, and pollen grains in GS2 with and without HS treatment. **(E, G)** Without HS treatment. **(F, H)** After HS treatment. Scale bars in **(C, D, G, H)** represent 100 µm. **(I)** Ratios of dead pollen grains. **(J)** Ratios of fertilized seeds. Significance level for *t*-test: **p < 0.01; ***p < 0.001.

Pollen grains from ZH11 and GS2 with and without HS treatment were observed and measured using a microscope after KI/I_2_ staining (**Figure 1**). The viability of pollen grains from both genotypes grown under control conditions was high. No significant difference was found between ZH11 and GS2. Dead pollen grains accounted for only 5.4% and 3.8% of pollen grains in ZH11 and GS2, respectively. The viability of pollen grains was decreased significantly by HS treatment in both genotypes. However, more severe heat-induced damage of pollen grains was found in GS2 anthers. The percentage of dead pollen grains was 34.6% in ZH11 after HS treatment, whereas this proportion increased significantly, to 62.6%, in GS2.

Next, we calculated the seed-setting rates of both genotypes. The seed-setting rates of ZH11 and GS2 under control conditions were 82.3% and 83.3%, respectively. The seed-setting rate was decreased significantly by HS in both genotypes. In addition, the rate of decrease was greater in GS2 than in ZH11, as we found that fertilized grains accounted for 35.4% of all grains in ZH11 after HS treatment, whereas only 21.1% of grains were fertilized in GS2 after HS treatment.

### 3.2 Overview of RNA-Seq profiles of anthers from the two genotypes under normal and HS conditions

**Figure 2:**
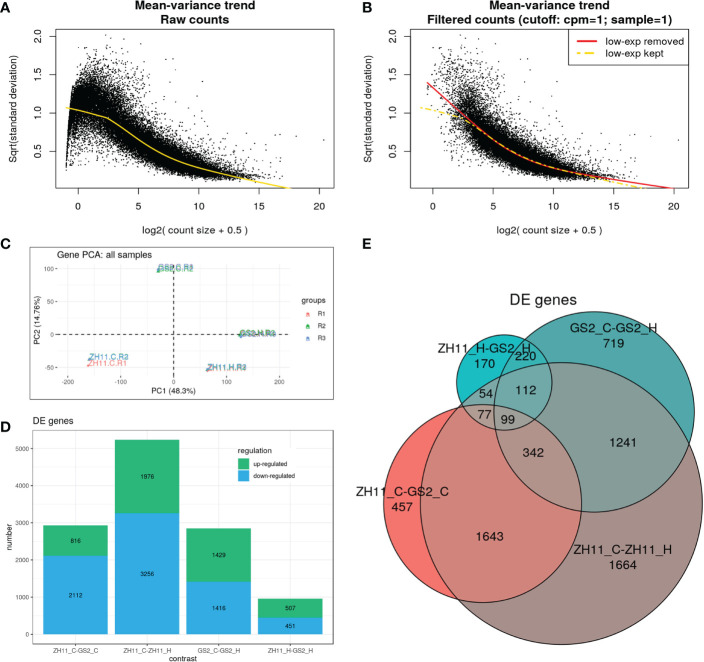
Gene expression patterns in Zhonghua 11 (ZH11) and grain size on chromosome 2 (GS2) under different temperature conditions. **(A)** Mean–variance trend of raw read counts (before filtering). **(B)** Mean–variance trend of filtered read counts (poorly expressed reads removed). **(C)** Principal component analysis (PCA) plots of 12 libraries. **(D)** Differentially expressed gene (DEG) numbers of four separate comparisons. The green box shows up-regulated genes and the blue box shows down-regulated genes. **(E)** Specific and overlapping DEG numbers across four different comparisons.

By running a RNA-Seq experiment with the collected anthers, we obtained 40–45 million raw reads from each of the 12 cDNA libraries, and at least 95.6% of raw reads from each library could be assembled to the reference sequence of the rice genome (**Supplementary Table 1**). In the 3D RNA-Seq analysis pipeline, low-count reads, that is, reads lower than one per million, and reads found only in one library, were removed from further analysis (**Figures 2A, B**). In principal component analysis, different samples separated from each other, while all three replicates from the same sample grouped together, suggesting high similarity between replicates (**Figure 2C**). The large distances between the ZH11 control group and other samples indicate that their transcriptomic profiles are very different. In contrast, the ZH11 and GS2 HS groups were close to each other, suggesting less transcriptomic difference between these two samples. Correspondingly, the analysis of DEG numbers showed that the numbers of down-regulated and up-regulated DEGs were, respectively, 2,112 and 816 in the comparison of the ZH11 and GS2 control groups; 3,256 and 1,976 in the comparison of the ZH11 control group and the ZH11 HS group; 1,476 and 1,429 in the comparison of the GS2 control group and the GS2 HS group; and 451 and 507 in the comparison of the ZH11 and GS2 HS groups (**Figure 2D**). Furthermore, the Venn diagram demonstrated that there were considerable numbers of overlapping and specific DEGs in different comparisons (**Figure 2E**).

### 3.3 Genes regulated by HS treatment in both ZH11 and GS2

**Figure 3:**
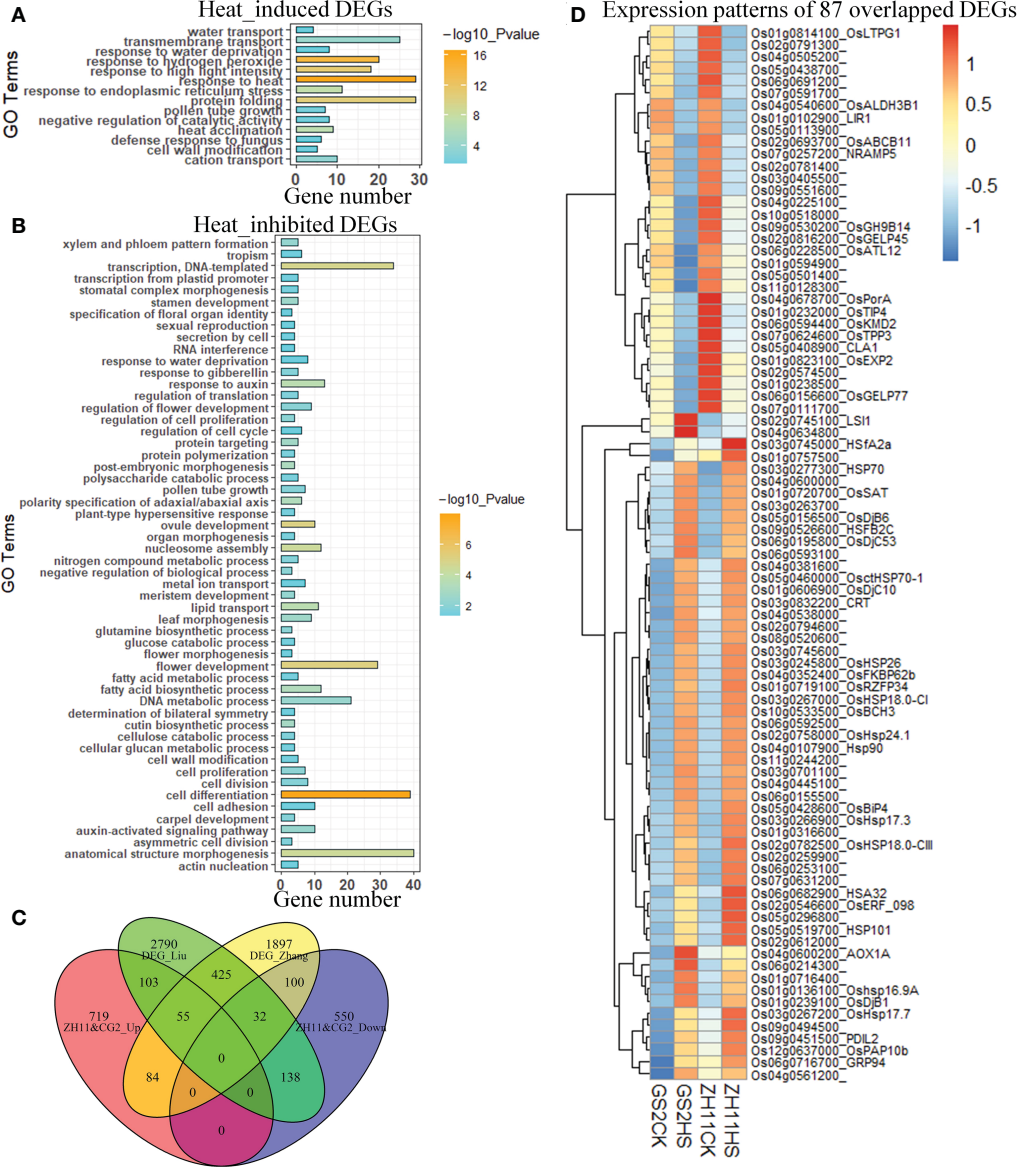
Heat-responsive differentially expressed genes (DEGs) in anthers from different genotypes. **(A)** Gene ontology analysis of genes up-regulated by heat stress (HS) in both Zhonghua 11 (ZH11) and grain size on chromosome 2 (GS2). **(B)** Gene ontology analysis of genes down-regulated by HS in both ZH11 and GS2. **(C)** Numbers of specific and overlapping DEGs in our experiment and in two other studies. **(D)** Expression of 87 genes (overlapping DEGs in three studies) in ZH11 and GS2 under normal and HS conditions.

Further analysis revealed a group of 961 genes that were up-regulated by HS in both ZH11 and GS2, and another group, of 820 genes, which were down-regulated in both varieties by HS (**Supplementary Tables 2, 3**). Gene ontology (GO) analysis revealed that HS up-regulated genes were enriched in the biological processes “response to heat”, “protein folding”, “response to hydrogen peroxide”, “response to high light intensity”, and so on (**Figure 3A**). On the other hand, HS-inhibited genes tend to be enriched in biological processes involved in cell differentiation and cell structure control, flower development, and gene transcription (**Figure 3B**). Inhibition of such genes explained why we observed a significant reduction in the viability of pollen grains from both genotypes.

Next, we employed two extra sets of RNA-Seq data,which have also been used to explore HS-induced transcriptomic changes in rice anthers (Zhang X. et al., 2012; Liu et al., 2020). Our analysis demonstrated that 103 and 84 genes from the commonly up-regulated DEGs in ZH11 and GS2, respectively, overlapped with one of the two RNA-Seq data sets employed, whereas another group of 55 DEGs overlapped with both data sets. In the case of HS-inhibited genes, 100, 138, and 32 genes from commonly down-regulated DEGs in ZH11 and GS2 were also found to be repressed in at least one of the employed data sets (**Figure 3C**). Further study showed that 40% of 55 commonly up-regulated genes in the three studies were enriched in the GO term of “response to heat”. This group of genes comprised, among others, *HSFA2a*, *Heat Shock Protein 101 (HSP101)*, *Heat Stress-associated 32kd protein (HSA32)*, and a group of *sHSP*s. In addition, a gene encoding a splicing factor, *RNA-binding Protein 1 (RNP1)* (Os01g031660), was found to be commonly up-regulated by HS in anthers from different rice genotypes. In contrast, another group of 32 genes were commonly down-regulated by HS treatment. These commonly suppressed genes included genes involved in cell division and flower development, such as an aquaporin protein (Os01g0232000), two GDSL-like lipase/acylhydrolases (Os02g0816200 and Os06g0156600), a lipid transfer protein (LTPL65, Os01g0814100), a transcription factor (Os02g0791300), a hormone-responsive gene (Os11g0128300), a core histone H2A (Os05g0113900), and a chloroplast precursor (Os04g0678700) (**Figure 3D**). These results suggest that these 87 genes may play important roles in the HS response of anthers from different rice genotypes.

### 3.4 Genes that are differentially expressed in ZH11 and GS2 after HS treatment

**Figure 4:**
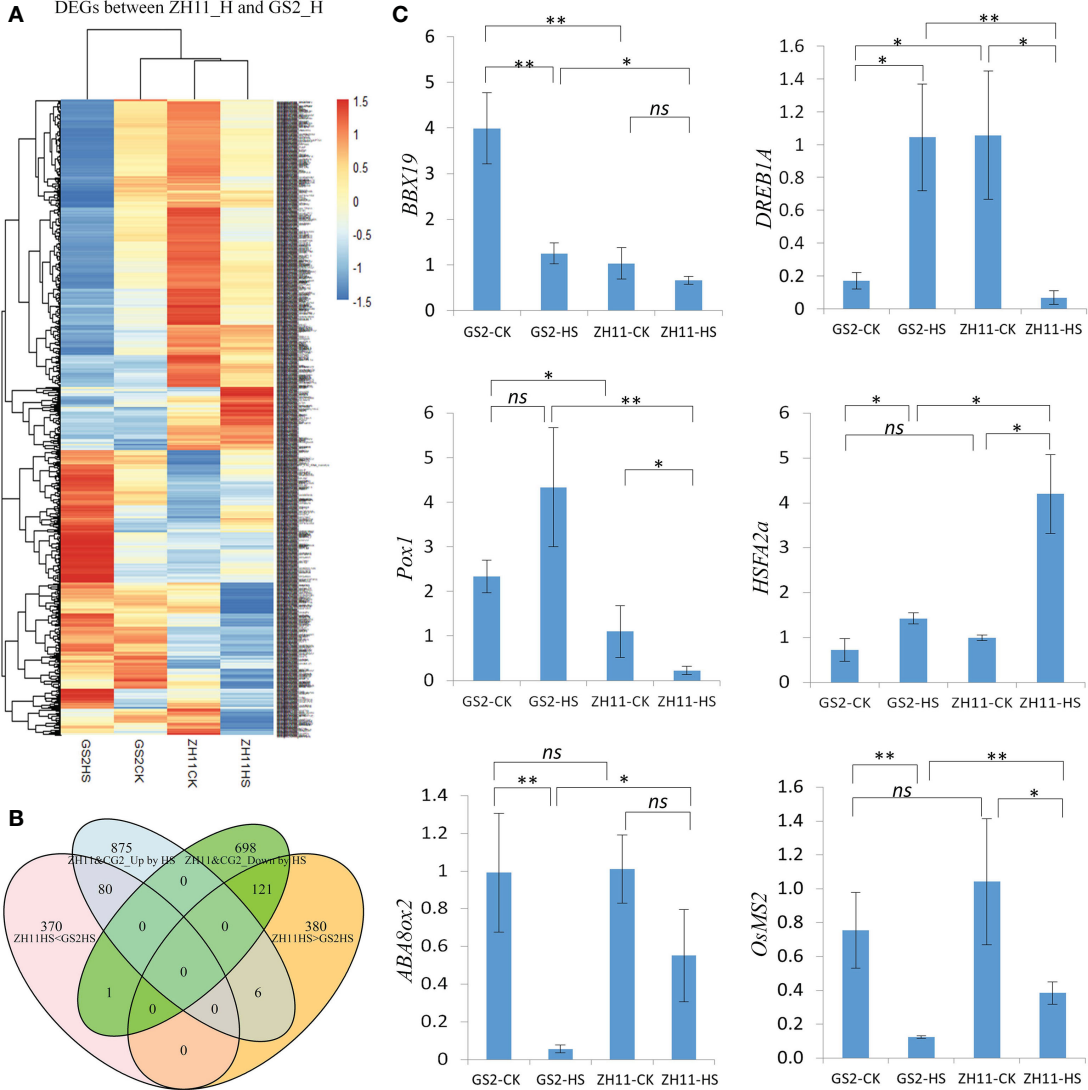
Expression patterns of differentially expressed genes (DEGs) between Zhonghua 11 (ZH11) and grain size on chromosome 2 (GS2) after heat stress (HS). **(A)** A heatmap showing expression patterns of DEGs in ZH11 and GS2 anthers after HS treatment. **(B)** Numbers of overlapping and specific DEGs between four comparisons in our study, namely ZH11HS < GS2HS (DEGs with lower expression levels in ZH11 than in GS2 after HS), ZH11&GS2_Up by HS (DEGs up-regulated by HS treatment in both ZH11 and GS2), ZH11&GS2_Down by HS (DEGs down-regulated by HS treatment in both ZH11 and GS2), and ZH11HS > GS2HS (DEGs with higher expression levels in ZH11 than in GS2 after HS). **(C)** Reverse transcription-quantitative PCR (qRT-PCR) validation of RNA sequencing (RNA Seq) data with some of these genes: *BBX19*, *DREB1A*, *Pox1*, *HSFA2a*, *ABA8ox2*, and *OsMS2*. Significance level for *t*-test: *p < 0.05; **p < 0.01. ns, non-significant.

As it has been found that the number of dead pollen grains in anthers after high-temperature treatment is greater in GS2 than in ZH11, we next aimed to uncover the transcriptomic difference between anthers from the two genotypes after HS. A heatmap comparing the patterns of expression of 958 DEGs in GS2 and ZH11 after HS treatment is shown in **Figure 4A**. The heatmap shows that the heat-mediated regulatory patterns of many genes were similar in the two genotypes. However, the same genes were expressed at different levels after HS treatment, resulting from either originally different levels under normal temperature conditions or different intensities of regulation between the two genotypes by HS treatment, or both. Interestingly, HS-mediated gene expression changes seemed to be more marked in GS2. After HS treatment, 80 HS-induced DEGs had higher levels of expression in GS2 than in ZH1, whereas 121 heat-suppressed DEGs were, in contrast, expressed at lower levels in GS2 than in ZH11 after HS treatment; one gene from this category was expressed at lower level in ZH11 than in GS2 after HS treatment (**Figure 4B**).

The change trends of genes detected from RNA-Seq data could be validated through qRT-PCR. Genes whose regulation under HS showed the same trend in ZH11 and GS2 included a B‐box containing protein *BBX19*, *HSFA2a*, *MALE STERILITY 2 (MS2)*, and *ABA 8′-Hydroxylase (ABA8ox2)* (**Figure 4C**). Regulation of some other genes under HS showed opposing trends in ZH11 and GS2. For example, *Dehydration Responsive Element Binding Protein 1A (DREB1A)* and *Peroxidase 1* (*Pox1*, Os05g0499300) were induced in GS2, but inhibited in ZH11, after HS treatment.

### 3.5 Genes essential for the development of anthers and pollen grains are differentially affected in ZH11 and GS2

Under normal conditions, a group of genes with higher expression levels in ZH11 than GS2 was enriched in GO terms including “carbohydrate metabolic process”, “lipid metabolic process”, “lipid transport”, and “photosynthesis”. In contrast, genes expressed at higher levels in GS2 were highly enriched in biological processes involved in pollen tube germination and growth, pollination, cell growth, transmembrane transport, and negative regulation of catalytic activity (**Supplementary Figure 1**). However, we did not find a significant difference in pollen viability under control conditions.

**Figure 5:**
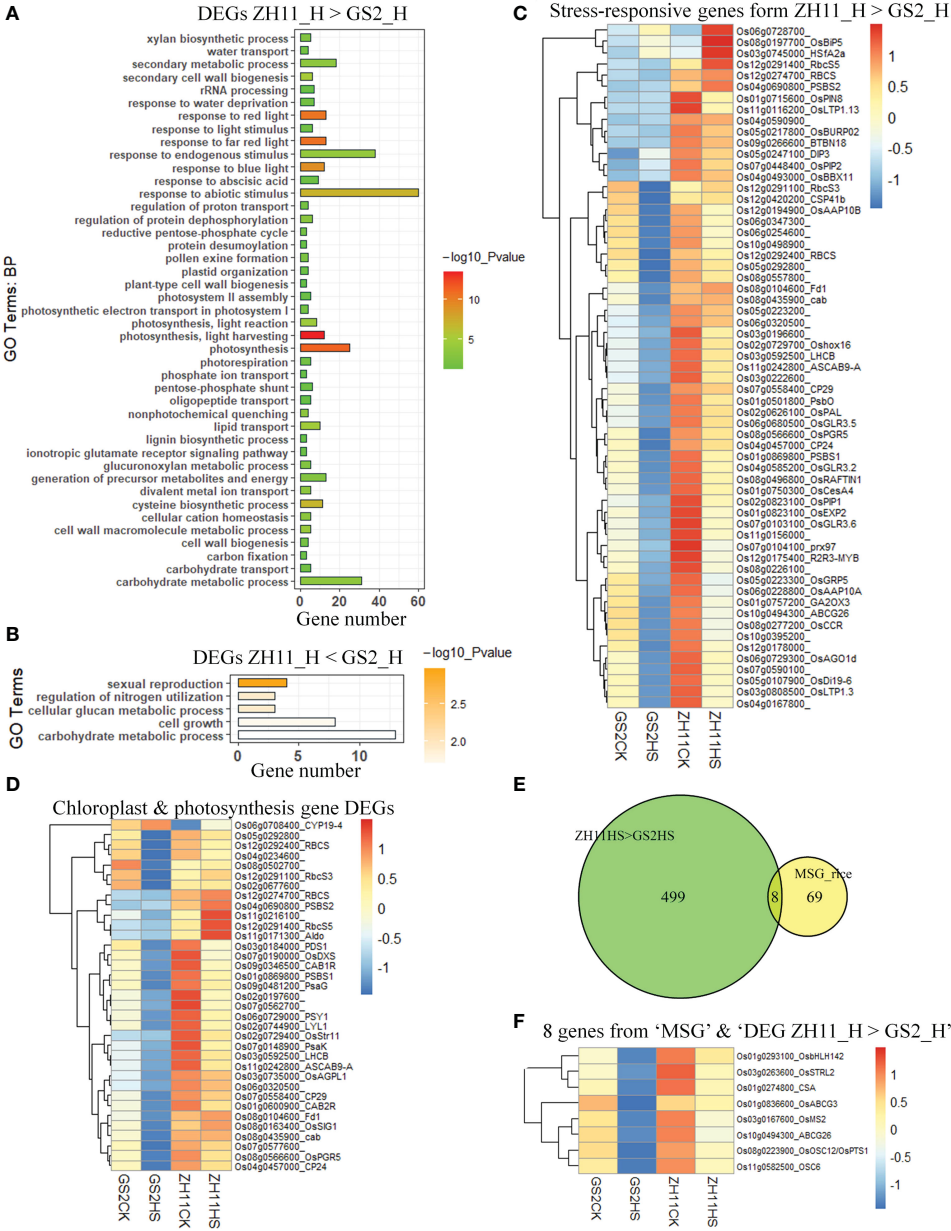
Differentially expressed genes (DEGs) essential for male fertility in anthers from Zhonghua 11 (ZH11) and grain size on chromosome 2 (GS2) after heat stress (HS) treatment. **(A)** Gene ontology (GO) analysis of DEGs with higher levels of expression in ZH11. **(B)** GO analysis of DEGs with lower levels of expression in ZH11. **(C)** Expression patterns of 60 stress-responsive DEGs with higher levels of expression levels ZH11. **(D)** Expression patterns of those DEGs involved in chloroplast development and photosynthesis. **(E)** The overlap of male sterile genes (MSGs) and those expressed at a higher level in ZH11 than in GS2. **(F)** Gene identifiers (IDs) and relative expression patterns of the eight MSGs in E.

Further analysis revealed that DEGs with higher levels of expression in ZH11 after HS treatment are involved in tens of biological processes (**Figure 5A**). About 60 DEGs are thought to play roles in response to abiotic stress, and 40 genes are involved in the response to endogenous stimuli. Interestingly, a group of DEGs with significantly higher levels of expression in ZH11 than in GS2 after HS treatment play important roles in chloroplast development and photosynthesis. In contrast, further GO analysis demonstrated that DEGs with lower levels of expression in ZH11 than in GS2 after HS treatment were greatly enriched, and thus with relatively low numbers, in five biological processes (**Figure 5B**), implying that the biological processes these genes are involved in have not yet been identified.

In fact, a great number of DEGs involved in chloroplast and photosynthesis are also responsive to abiotic stress (**Figures 5C, D**). Only a few abiotic stress-responsive genes, such as *HSFA2a* and *OsBiP5*, were up-regulated by HS, whereas most of the remaining genes were down-regulated in both genotypes by HS treatment (**Figure 5C**). Similarly, many genes related to photosynthesis were also down-regulated in both genotypes. However, these photosynthesis genes were down-regulated to different levels in ZH11 and GS2 by the same HS treatment. All genes, except *CYP19-4*, were expressed at higher levels in ZH11 than in GS2 after HS treatment (**Figure 5D**). Given that the abundance of dead pollen grains was greater in GS2 than in ZH11 after HS, we supposed that sustaining the expression of these genes at relatively high levels might contribute to reduced heat-induced damage to rice anthers.

Next, we investigated a group of genes that have been identified as male sterile genes (MSGs) in rice, the mutation of which would cause total or partial male sterilization in rice [as reviewed by Abbas et al. (2021)]. Interestingly, 8 out of 77 MSGs were expressed at higher levels in ZH11 than in GS2 after HS treatment (**Figure 5E**). In contrast, no MSGs were expressed at significantly higher levels in GS2 than in ZH11 after the same HS treatment. The eight MSGs with higher levels of expression in ZH11 than in GS2 after HS were five genes involved in lipid metabolism [i.e., *OsC6* (Os11g0582500), *OsABCG26* (Os10g0494300), *OsDPW* (Os03g0167600), *OsSTRL2* (Os03g0263600), and *OsABCG3* (Os01g0836600], a bHLH transcription factor [*TIP2/TDR/bHLH142* (Os01g0293100)], an R2R3 MYB transcription factor *Carbon Starved Anther (CSA, *encoded by Os01g0274800), and a triterpene synthase [*OsC12/PTS1* (Os08g0223900)] (**Figure 5F**). *OsC6* was also one of the top 50 DEGs with much higher expression levels in ZH11 than in GS2 after HS treatment (**Supplementary Figure 2**).

### 3.6 Expression of the GRF gene family under different temperature conditions in GS2 and ZH11

**Figure 6:**
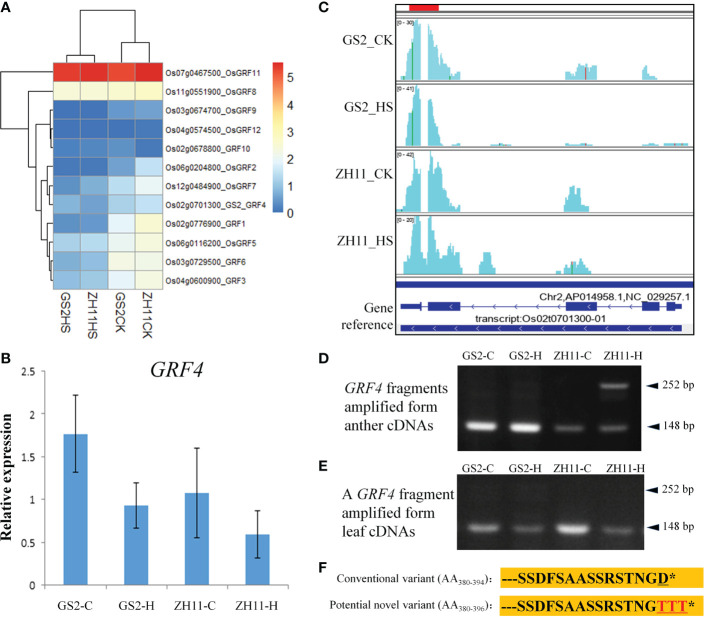
Expressions of growth regulation factors (GRFs) under different temperature conditions. **(A)** Expression patterns of GRFs in RNA sequencing (RNA-Seq) data. **(B)** Expression of *OsGRF4* (*Oryza sativa* growth-regulating factor 4) measured by reverse transcription-quantitative PCR (qRT-PCR). **(C)** Visualization of coverage of sequencing reads annotated to *OsGRF4* gene using the IGV program. **(D)** Reverse transcription-PCR (RT-PCR) validation of the intron retention (IR) event detected in *OsGRF4*. **(E)** RT-PCR amplification of *OsGRF4* by using leaf cDNA as a template. **(F)** Amino acid residues of the conventional OsGRF4 protein and that of the potential protein variant translated from the intron-retained mRNA isoform. Only amino acid residues from position 380 on are shown. * stands for the terminal point of the amino acid sequence.

As the difference between the two rice genotypes used here was thought to have resulted from a couple of nucleotide variations (1,187TC→AA) in a GRF, *OsGRF4*, we were curious to know the patterns of expression of this gene and of its family members in the anthers of the two genotypes. As shown in **Figure 6A**, among these 12 GRF genes, *OsGRF11* showed the highest level of expression in anthers, followed by *OsGRF8*, under either normal or high temperature. Almost all GRFs were suppressed by HS in both genotypes, except for *GRF10* and *GRF11*: the former was suppressed by HS in GS2 but induced in ZH11, whereas the latter was induced by HS in GS2 but suppressed in ZH11. The expression level of *OsGRF4* in anthers was lower than that of many other GRF genes. Furthermore, the transcripts of this gene seemed to accumulate less in ZH11 under HS conditions when tested by qPCR (**Figure 6B**).

When observing read coverages of *OsGRF4* using the IGV program, we found another sequence variation, in addition to the previously reported variation (1,187TC→AA). This novel variation is located at 3′-UTR of the last exon, where an ‘A’ was replaced by a ‘T’ in GS2 (as this variation was found in all GS2 genotype samples but not in any ZH11 genotypes, we suggest that it is not a random error caused by sequencing). More interestingly, we found that the pre-mRNA splicing patterns of *OsGRF4* were different in ZH11 and GS2 genotypes after HS, when read coverages of the target genes were visualized using the IGV program (**Figure 6C**). The last intron of this gene underwent intron retention (IR) in ZH11 after HS. This AS regulation was not found in any of the GS2 samples, either with or without HS treatment. In other words, the splicing patterns of the gene in the GS2 background remained the same as under the normal temperature condition. We further tested this AS event using RT-PCR. We found that an extra DNA fragment (252 bp) was amplified in ZH11 HS group samples (**Figure 6D**). This novel amplified fragment was predicted to be the intron-retained isoform owing to its size. Moreover, the AS of *OsGRF4* seemed to be carried out only in ZH11 anthers after HS treatment, as we could not detect the same AS event in leaves from either genotype under the same temperature conditions (**Figure 6E**). Bioinformatic analysis revealed that the resulting mRNA isoform of *OsGRF4* in ZH11 anthers induced a stop codon in the retained intron, which would induce RNA degradation if it triggered the nonsense-mediated degradation (NMD) system. Otherwise, the resulting mRNA isoform was predicted to be translated into a sequence-varied protein, with the last amino acid, D (Asp), at the C-terminal replaced by three contiguous T (Thr) residues (**Figure 6F**). Thus, the mechanism for the appearance of AS in *OsGRF4* is yet not clear, and determination of its biological significance for the heat tolerance of anthers and pollen grains in rice requires further observation.

### 3.7 Differential AS analysis between ZH11 and GS2 after HS treatment

**Figure 7:**
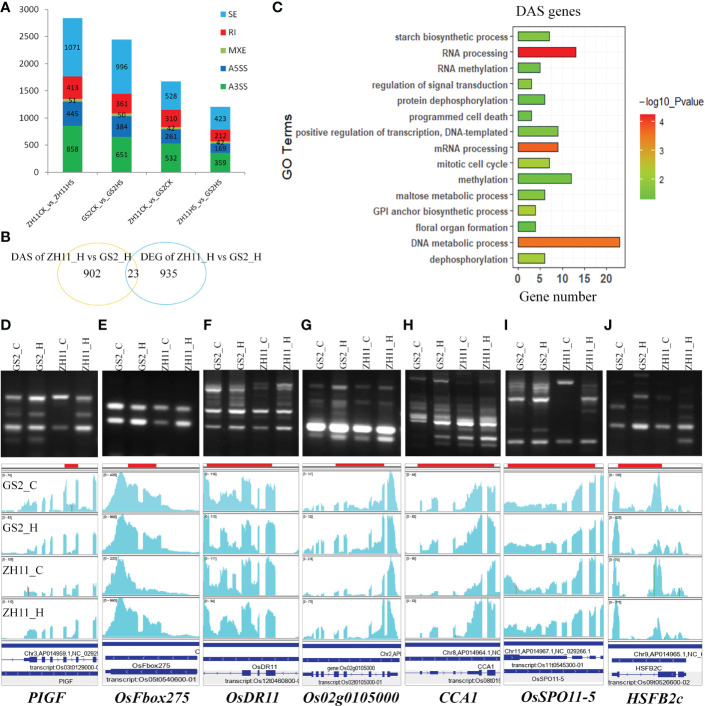
Detection of differential alternative splicing (DAS) genes in Zhonghua 11 (ZH11) and grain size on chromosome 2 (GS2). **(A)** Number of DAS events in four different comparisons. **(B)** Overlap of differentially expressed genes (DEGs) and DAS genes in the comparison of the ZH11 HS group and the GS2 HS group. **(C)** Gene ontology (GO) analysis of DAS genes in the comparison of the ZH11 HS group and the GS2 HS group. **(D–J)** Alternative splicing (AS) events on seven genes as determined through reverse transcription-PCR (RT-PCR) (upper) and the IGV program (bottom).

Next, we employed a widely used pre-mRNA splicing analysis program, rMATS, to explore AS events in the RNA-Seq samples. As shown in **Figure 7A**, AS events were defined as a skip exon (SE), a retained intron (RI), mutually exclusive exons (MXE), an alternative 3′ splice site (A3’SS), and an alternative 5′ splice site (A5’SS) in our analysis. There were 2,838, 2,442, 1,673, and 1,205 significant differential AS (DAS) events in comparisons of ZH11 control group vs. ZH11 HS group, GS2 control group vs. GS2 HS group, ZH11 control group vs. GS2 control group, and ZH11 HS group vs. GS2 HS group, respectively. SE and A3′SS events accounted for more than two-thirds of the total AS events in each comparison, with IR and A5′SS events the next most frequent. Interestingly, only 23 genes were considered to be DEGs and also DAS genes across ZH11 and GS2 after HS, that is, less than 3% of these 1,960 disturbed genes were regulated together at transcriptional and splicing levels (**Figure 7B**). GO analysis revealed that DAS genes tend to be highly enriched in nucleic acid-binding processes, such as DNA metabolism, RNA processing, gene transcription, etc. A group of genes involved in pollen grain development could also be differentially spliced. As we found that DAS genes were also enriched in GO terms, such as “starch biosynthetic process”, “mitotic cell cycle”, and “floral organ formation” (**Figure 7C**).

We randomly picked up several genes that was considered to be differently spliced in ZH11 and GS2 under heat stress conditions, and validated them through RT-PCR. It was demonstrated that encoding genes of important regulators, such as *CCA1*, *OsSPO11*, *HSFB2c*, and *OsDR11*, could amplify more than one target band (**Figures 7D–J**), suggesting that different mRNA isoforms could be generated after the pre-mRNA splicing process. Moreover, the intensities of amplified fragments of the same genes varied between ZH11 and GS2 after HS, suggesting different abundances of alternatively spliced mRNA isoforms in these two samples, which corresponds to snapshots from the IGV program (shown below each RT-PCR gel panel). Notably, not all AS events detected through RT-PCR and the IGV program were considered significant DAS events in the rMATS program. Reasons for this gap could be the relatively low read coverage of AS events in RNA-Seq data or the technical preference of the data analysis program (i.e., rMATS focusing on variations of junction counts between different exons, but not IR (Shen et al., 2014).

## 4 Discussion

A two-nucleotide substitution in the *OsGRF4* gene could enhance NUE and carbohydrate accumulation under normal conditions, ultimately resulting in enhanced grain size and yields (Che et al., 2015; Duan et al., 2015; Li et al., 2018). The meiotic stage is one of the developmental phases that is most sensitive to HS (Chen et al., 2016). Our experimental results demonstrate that heat-induced damage in anthers was more severe in the near-isogenic line GS2 (*OsGRF4* variation line) than in its wild-type background ZH11. This indicates a potential risk of more severe HS-induced productivity loss from this rice genotype if planted in areas that are frequently exposed to high temperatures and heatwaves during the meiotic stage.

The involvement of GRF family proteins in stress responses has been found before in plants. For example, it has been found that MYB61 is regulated by OsGRF4, and promotes nitrogen utilization and biomass production in rice (Gao et al., 2020). Thus, we did not find differential expression of MYB61 from our RNA-Seq data here, suggesting that there could be other downstream pathways involved in differentiated carbon and nitrogen metabolisms in rice. In *Arabidopsis*, a mutation of AtGRF7 enhances its drought and salt stress tolerance, because AtGRF7 suppresses the expression of stress-responsive genes and then decreases the stress resistance in wild-type plants (Kim et al., 2012). Transgenic seedlings of a hybrid poplar (i.e., *Populus alba* × *Populus glandulosa*) overexpressing a specific GRF15 mRNA lacking target sites of miR396a exhibited enhanced heat tolerance and photosynthetic efficiency (Zhao et al., 2021), suggesting that GRF15 plays an important role in responding to HS in poplar seedlings. However, it is still unclear whether or not, in this transgenic tree, the heat tolerance of the flowers as well of the vegetative tissues was enhanced.

GO analysis demonstrated that several biological processes, including “response to heat”, “protein folding”, “response to high light intensity”, and “response to hydrogen peroxide”, were highly enriched in both genotypes in response to high-temperature stress treatment. This result is similar to what had been found recently in wheat anthers (Browne et al., 2021). In this study, a great number of heat-responsive genes, including *HSFs* and *HSPs*, were up-regulated by HS in anthers from both genotypes. Heat induction of such genes was found in different organisms, and at different developmental stages of plants (McClellan et al., 2007; Gonzalez-Schain et al., 2016; Ling et al., 2018), suggesting that all of these genes play a role in facilitating heat tolerance in different plant tissues. In total, 87 genes are regulated in the anthers of both ZH11 and GS2, as well as in the rice genotypes “996”, “SDWG005”, and “9311” (Zhang X. et al., 2012; Liu et al., 2020). These common up-regulated DEGs include not only *HSFs* and *HSPs*, but also two pre-mRNA splicing factors, *SF2* (Os01g0316600) and *PRP4* (Os03g0701100), suggesting that they would function as regulators of pre-mRNA splicing under HS conditions.

Abiotic stress can alter the composition of core histone H2A and chromatin remodeling factors. It has been suggested that H2A.Z nucleosomes wrap DNA more tightly under low temperature while more loosely under high temperature, which influences the ability of RNA polymerase II to transcribe genes in response to temperature variation (Kumar and Wigge, 2010). Here, we found that the DEGs that were down-regulated in anthers from different rice genotypes included not only those involved in cell division and flower development, but also genes encoding core histones H2A and H3, SWI/SNF-related chromatin-binding proteins, HMG1/2 (Os09g0551600, and THION27 (Os01g0594900). Given that these genes may function in processes, such as preservation of membrane integrity and chromosome architecture, suppression of their expression was assumed to be a reason for the developmental deficiency of pollen grains after HS treatment.

Reduction and/or alteration in carbohydrate reserves causes developmental defects in pollen grains and male sterility under stress conditions (De Storme and Geelen, 2014). The transcriptomic analysis in our study revealed that, in addition to genes involved in abiotic and endogenous stimuli, genes involved in chloroplast development and photosynthesis and carbohydrate metabolism accounted for the majority of DEGs that after HS treatment were expressed at higher levels in the anthers of ZH11 than in those of GS2. Almost all of chloroplast- and photosynthesis-related genes were expressed at lower levels in GS2 than in ZH11 after HS treatment. More severe heat inhibition of photosynthesis-related genes is associated with a greater decrease in pollen grain viability in GS2 anthers. Although photosynthetic activity seems to be carried out mainly in the leaves of plants, several recent studies have confirmed that properly expressions of photosynthesis and chloroplast genes are essential for anther development in maize (Murphy et al., 2015; Zhu et al., 2020), petunias (Yue et al., 2018), wheat (Liu et al., 2018), and rice (Basnet et al., 2019). Nearly all (> 96%) photosynthesis-associated genes found in the maize leaf were expressed in most subepidermal and endothecial cells of the anthers, although no photosynthetic light reaction activity from the anthers was detected *in vitro* (Murphy et al., 2015). In wheat anthers, it was found that HS caused significant down-regulation of chloroplast-related genes together with the disturbance of starch and sucrose metabolism, and could ultimately result in severe decease of pollen fertility (Liu et al., 2018). By comparing gene expression profiles in anthers from wild-type rice and from *phyA* and *phyB* single and double mutants, it was found that carbohydrate metabolism and stress- and photosynthesis-related genes were significantly affected in the *phyA phyB* double mutant (Basnet et al., 2019), indicating a complex regulatory network underlying phytochrome-mediated anther and pollen development in rice. Another study of rice anthers demonstrated dynamic change in the expression of chlorophyll- and photosynthesis-related genes, which were up-regulated immediately after HS and then suppressed when the stress was prolonged (Liu et al., 2020).

More severe HS-induced male sterility in GS2 could be further attributed to more vulnerable lipid metabolic activity in anthers, as five of the eight MSGs that are differentially expressed in the anthers of GS2 and ZH11 are lipid metabolism genes, and all are consistently expressed at significantly lower levels in GS2 anthers than in ZH11 anthers. In support of this, premature programed cell death of tapetal cells was found from maize anthers with a mutation in *ZmGPAT6*, a male sterile gene that encodes a glycerol-3-phosphate acyltransferase. *ZmGPAT6*-mediated lipid biosynthesis maintains starch metabolism and photosynthetic activities of endothecium (En) chloroplasts, which are crucial for maize anther development (Zhu et al., 2020).

A more severe HS-induced abortion phenotype of GS2 is correlated with greater inhibition of the critical transcription factors necessary for pollen grain development and fertilization. *CSA* is a gene that encodes a MYB domain protein regulating sugar partitioning for rice pollen development (Zhang et al., 2010), and controls the development of the reverse photosensitive genic male sterile (rPGMS) trait (i.e., exhibiting male fertility under long-day conditions and male sterility under short-day conditions) in japonica rice (Zhang et al., 2013; Zhu et al., 2015). This gene was down-regulated to a significantly lower level in GS2 anthers under HS conditions. A comparative study in rice found that *OsHSFA2a* was induced significantly in anthers, and its induction intensity was positively correlated with the heat tolerance of anthers (Gonzalez-Schain et al., 2016). It has been reported that mutation in the *bHLH142* gene also causes complete male sterility (Ranjan et al., 2017; Abbas et al., 2021).

High temperatures target not only gene transcription, but also mRNA processing, in anthers and pollen grains. Global AS can be triggered by HS in different organisms (Ling et al., 2021) and in this study the number of AS events detected differed significantly between different genotypes and under different temperature conditions. Thus, the most frequent AS type detected here through rMATS was exon skipping (ES); this is inconsistent with previous reports, which suggest IR is the most prevalent AS type carried out in plants (Jiang et al., 2017; Ling et al., 2018). The divergence can likely be attributed to different preferences between AS analysis programs, as we know that rMATS was originally designed for detecting AS events in mammal cells, where ES is the predominant type of AS (Shen et al., 2014). We detected a great numbers of IR events using the IGV program coupled with RT-PCR validation, but not in rMATS, suggesting that considerable numbers of novel IR events or those with relatively low intensity could be ignored by this program. Nevertheless, many IR events were detected in genes encoding stress regulators and also in those genes critical for gametophyte development, including *HSFB2c*, *CCA1*, *OsSPO11-5*, and *OsDR11*.

More interestingly, *OsGRF4* itself underwent IR specifically in anthers from ZH11 under the given HS conditions. There could be several reasons for this genotypic-specific AS event. The first reason could be the specific AS regulated by variation of *cis*-elements such as sequence changes in the *OsGRF4* gene between ZH11 and GS2 backgrounds. Second, differentiated expression of splicing factors could also affect the pre-mRNA splicing process. Third, different cellular states, such as different carbon and nitrogen supply, may also impact the pre-mRNA splicing patterns. In fact, some clues can be obtained from recent studies. A great number of genes encoding splicing factors, miRNAs, long non-coding RNAs (lncRNAs), and transcription factors were found to undergo AS in young panicles and florets of rice (Li et al., 2021). The same genes from different rice genotypes carried out differential AS regulations in response to salt stress stimulation (Jian et al., 2022). Interestingly, not only growth conditions but also nutrition status affect AS regulation of related genes in different plants (Zhang Y. M. et al., 2012; Li H. et al., 2016; Nishida et al., 2017; Wang et al., 2019). For example, nutrient stress caused differential AS regulation of the *NRR* gene in rice (Zhang Y. M. et al., 2012). Moreover, a recent study demonstrated that nitrogen starvation modulated differential AS and transcript usage of various metabolism-related genes in rice (Chaudhary and Kalkal, 2021), and AS of *OsGS1;1* affects NUE, grain development, and amylose content in rice (Liu et al., 2022).

In conclusion, under HS conditions, the rice variety GS2, containing *OsGRF4^AA^*, caused the alteration of nutrition allocation and metabolism in reproductive tissues, which could be mediated by, and also feed back negatively to, transcriptional and splicing regulation of essential genes. The combined impact made GS2 anthers and the developing male gametophytes more sensitive to HS.

## Data availability statement

The data sets presented in this study can be found in SRA repositories https://www.ncbi.nlm.nih.gov/bioproject/PRJNA915644. The names of the repository/repositories and accession number(s) can be found in the article/**Supplementary Material**.

## Author contributions

YL and DZ had the idea, organized the whole program, and modified the manuscript. YM and GL ran most of the experiments, done the data analysis, and wrote the manuscript. LL, YZ, JL, MY, SC, and QL participated in some experiments and data analysis. GF provided suggestions for the experiments. All authors contributed to the article and approved the submitted version.

## Supplementary material

Supplementary Figure 1 Biological process enrichment of differentially expressed genes (DEGs) between Zhonghua 11 (ZH11) and grain size on chromosome 2 (GS2) under normal conditions. **(A)** DEGs with higher expressional levels in ZH11. **(B)** DEGs with higher expression levels in GS2.

Supplementary Figure 2 Top 50 differentially expressed genes (DEGs) in comparisons of ZH11 HS group > GS2 HS group **(A)** and ZH11 HS group < GS2 HS group **(B)**.
