# Supplementary figures and images for: OsGRF4AA compromises heat tolerance of developing pollen grains in rice
Source: Front Plant Sci. 2023 Feb 22;14:1121852. doi: 10.3389/fpls.2023.1121852 (PMC9992635; doi:10.3389/fpls.2023.1121852)

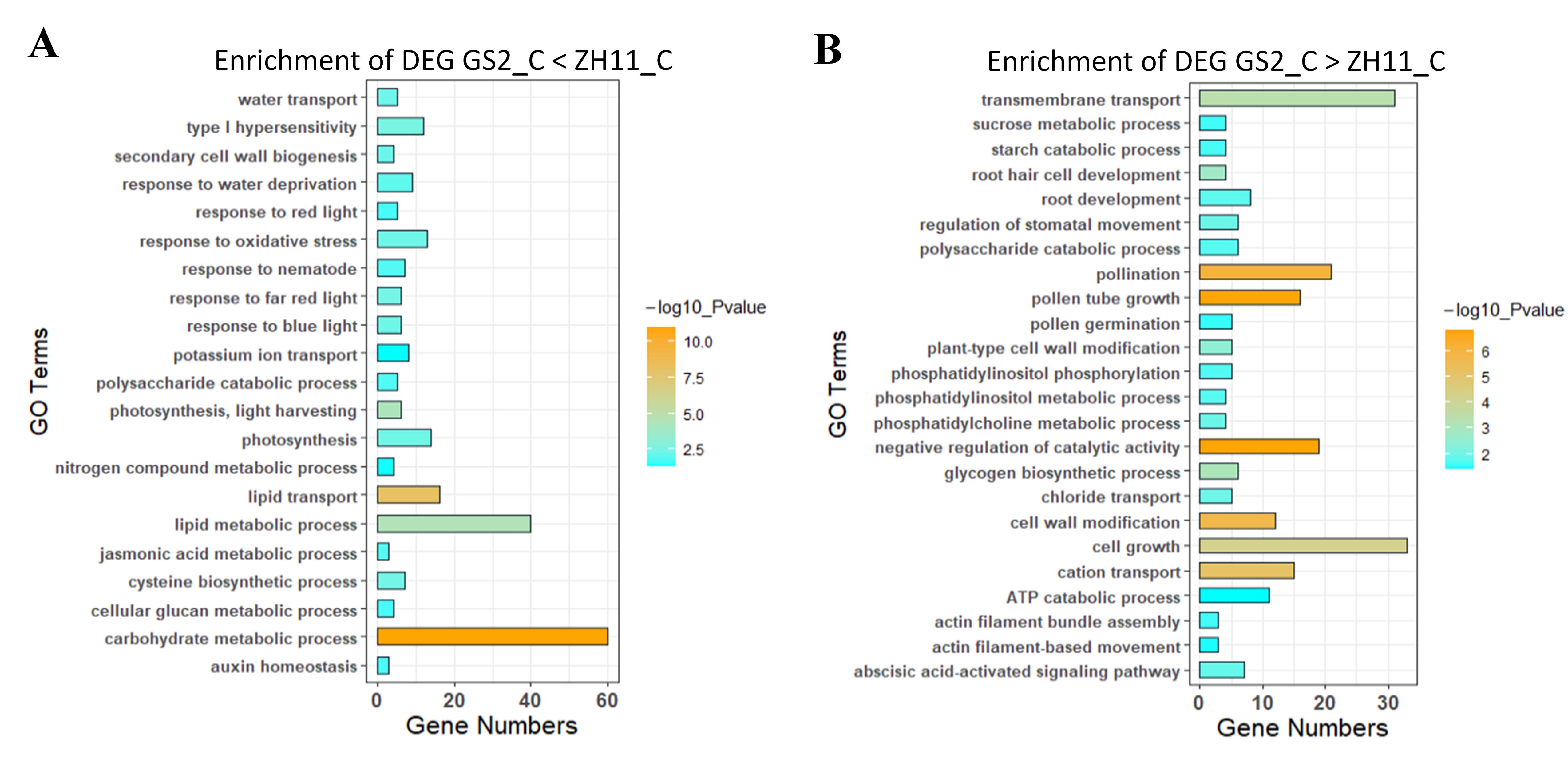

Supplement: Supplementary Figure 1 — Biological process enrichment of differentially expressed genes (DEGs) between Zhonghua 11 (ZH11) and grain size on chromosome 2 (GS2) under normal conditions. (A) DEGs with higher expressional levels in ZH11. (B) DEGs with higher expression levels in GS2. [file Image_1.jpeg]

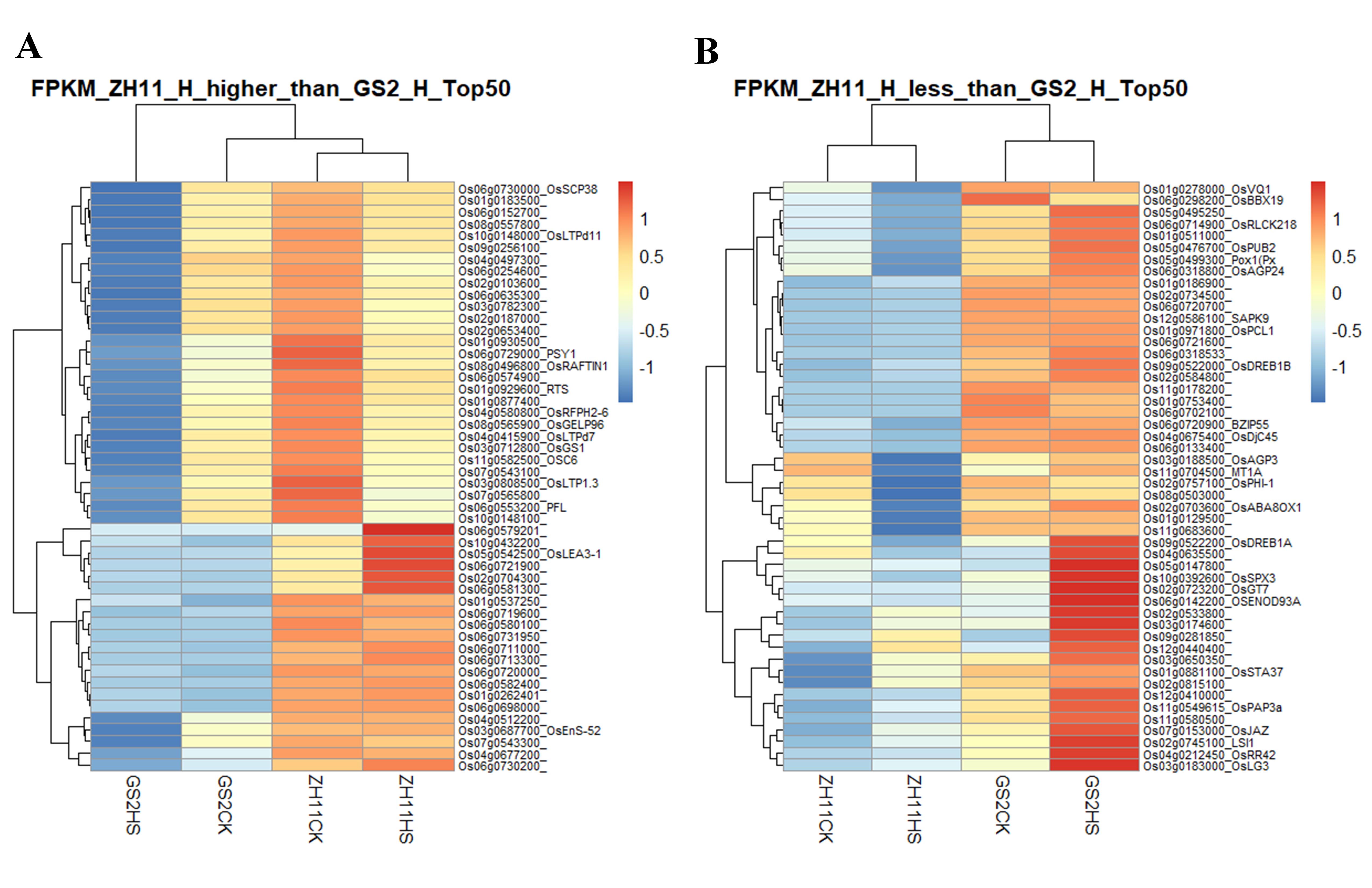

Supplement: Supplementary Figure 2 — Top 50 differentially expressed genes (DEGs) in comparisons of ZH11 HS group > GS2 HS group (A) and ZH11 HS group < GS2 HS group (B). [file Image_2.jpeg]
